# Do maturation, anthropometrics and leg muscle qualities influence repeated change of direction performance in adolescent boys and girls?

**DOI:** 10.5114/biolsport.2023.123322

**Published:** 2023-03-06

**Authors:** Matteo Giuriato, Vittoria Carnevale Pellino, Nicola Lovecchio, Roberto Codella, Matteo Vandoni, Scott Talpey

**Affiliations:** 1 Department of Human Science, University of Verona, Verona, Italy; 2 Gdansk University of Physical Education and Sport, Gdańsk, Poland; 3 Laboratory of Adapted Motor Activity (LAMA), Department of Public Health, Experimental Medicine and Forensic Science, University of Pavia, Pavia, Italy; 4 Industrial Engineering, University of Tor Vergata, Rome, Italy; 5 Department of Human and Social Sciences, University of Bergamo, Bergamo, Italy; 6 Department of Endocrinology, Nutrition and Metabolic Diseases, IRCCS MultiMedica, Milano, Italy; 7 Department of Biomedical Science for Health, Università degli Studi di Milano, Milano, Italy; 8 Institute of Health and Wellbeing, Federation University Australia, Ballarat Australia; 9 School of Health and Human Services, Southern Connecticut State University, New Haven, CT, USA

**Keywords:** Change of Direction, Speed, Adolescent, Eurofit Test, Peak Height Velocity

## Abstract

The ability to change direction rapidly is a key fitness quality especially in invasive sports where young players perform approximately 300 changes of direction in a game. There is currently limited understanding of how anthropometric characteristics and maturation status influence change of direction ability in adolescent. Therefore, the purpose of this investigation is to assess the influence of anthropometrics and maturation status on change of direction ability in young people. The study involved 706 adolescents (367 girls) aged 14–19-year-old attending the same high school in Northern Italy. Stature, body mass, seated height and leg length were measured to determine the anthropometrics and maturation status of the participants. Repeated change of direction ability (10 × 5 m shuttle run test), lower limb power and muscle strength were evaluated using field tests from the Eurofit test battery. Maturity offset was calculated separately for boys and girls, in accord with the equation proposed by Mirwald. Preliminary analysis with 10 × 5 m as a dependent variable and sex and PHV as a fixed factor, suggests a significant difference between sex (p < 0.001; d = 0.35) but not with PHV (p = 0.986; d = 0.000) and interaction PHV × sex (p = 0.836; d = 0.000). Our results suggested that repeated change of direction performance was influenced by anthropometrics, maturation and muscle qualities in adolescent boys and girls.

## INTRODUCTION

The ability to change direction (COD) rapidly is a key physical fitness component for many invasive sports [1]. Moreover, an athlete’s ability to change direction effectively provides the technical underpinning for their agility which is the ability to produce an accurate and effective change of direction in response to a stimulus [2]. Change of direction ability has been shown to possess discriminative ability between higher and lower-level youth performers in invasive team sports [3] and individual sports [1, 4]. Changes of direction are performed frequently in many sports [5, 6]. In fact, it has been recently noted that youth soccer players perform approximately 300 changes of direction in a game [7]. A successful COD involves a quick deceleration and a coordinated repositioning of the body prior to a re-acceleration in a new direction [7]. An individual’s technical ability, lower limb strength capabilities, and sprinting speed are noted underpinning factors of COD ability [4, 7, 8].

Owing to of the complexity associated with growth and maturation the development of COD ability in youth is highly variable [9]. As a young person matures, changes in limb length and body composition can significantly influence movement quality [10, 11]. For example, shuttle run and 30 m sprint performance have been found to significantly improve at the moment of peak height velocity (PHV) occurrence in a large cohort of youth soccer players [11]. Due to the variable nature of maturation, theoretical models of exercise prescription in youth have proposed “windows of opportunity” where specific physical qualities can be emphasized at appropriate time associated with a young person’s maturation status [9, 12]. Within these models’ it is postulated that the development of COD ability should be targeted at an individual’s PHV alongside the development of strength, speed and power [12].

Evidence to support these ‘windows of opportunity’ are well established within the literature for the development of strength and endurance in youth [13, 14, 15]. However, there is currently limited understanding of how anthropometric characteristics (e.g., height, leg length, waist circumference) and maturation status influence COD ability in young people. Therefore, the purpose of this investigation is to assess the influence of anthropometric measures and maturation status on COD ability in young people. Clarity on the interplay between COD ability and a young person’s anthropometrics and maturation status will help practitioners such as Physical Education (PE) teachers, coaches and trainers understand when and how to best prescribe exercise to improve this valuable physical fitness component.

## MATERIALS AND METHODS

### Participants

The study involved 706 adolescents (367 girls) aged 14–19-year-old attending the same high school in Northern Italy.

All the subjects were healthy (in possession of a medical certificate of good health status) injury free and did not possess and neurological disorders. All outcome measures were collected during Physical Education classes as part of routine fitness testing with the Eurofit test battery.

A cross-sectional cohort design was incorporated in the study and provides separate descriptive measures for both boys and girls. This study was approved by the Ethics Committee at Regional School (Lombardia), (number: “UP 1819-15”) in Italy. Written information of the study, aim and procedures was given to teachers and students. An informed consent was obtained from all participant’s parents or legal guardians.

### Procedure

All the tests were conducted by a team of 5 students enrolled in the Sports Sciences Master’s during the period of the PE program [16]. To increase reliability and validity each assessor undertook one week of training for the specific test that were administrating. Previous determination of the reliability of the assessor was performed to ensure accuracy and repeatability of the procedure (inter- and intra- examiner ICC of 0.96 and 0.98, respectively).

The school’ PE teacher was present during test administration to ensure that students participating in the investigation complied with the procedures.

Anthropometric (body mass, height, sitting height, leg length) and physical fitness assessments (three field tests) of the adolescents were completed over a four week period with a one round of measurements obtained per week to avoid fatigue. All the participants were free to withdraw their participation at any time. Anthropometrics characteristics were evaluated 1 day before the performance test. The order of the test battery was as follows, 10 × 5 m test, sit up test, and finally the standing broad jump. Between each test the subjects had a five-minute rest to facilitate recovery.

### Anthropometric measures

Anthropometric characteristics were taken by trained operators (quality-control coefficient for inter-and intra-observer reliability, 95% confidence interval). Standing height was evaluated to the nearest 0.5 cm (Seca Stadiometer 208) without shoes, feet together, and head in the Frankfort plane. Body mass was measured to the nearest 0.5 kg (Seca Beam Balance 710), with participants wearing minimal clothing [17, 18].

### Measure of Maturity

Maturity off set was calculated separately for boys and girls, in accord with the equation proposed by Mirwald et al. [19].

In boys the predictive equation was as follows: Maturity Offset = -29.769 + 0.0003007 · Leg Length and Sitting Height interaction + 0.01177 · Age and Leg Length interaction + 0.01639 · Age and Sitting Height interaction + 0.445 · Leg by Height ratio, where R = 0.96, R^2^ + 0.915, and SEE = 0.490 [19].

In girls, the predictive equation was Maturity Offset = -16.364 + 0.0002309 · Leg Length and Sitting Height interaction + 0.006277 · Age and Sitting Height interaction + 0.179 · Leg by Height ratio + 0.0009428 · Age and Weight interaction, where R = 0.95, R^2^ = 0.910, and SEE = 0.499 [19].

Corresponding statistics (except for actual offset) were calculated relative to years before/after observed Peak Height Velocity (PHV): -3 = -2.51 to -3.50, -2 = -1.51 to -2.50, -1 = -0.51 to -1.50, 0 = -0.50 to +0.49, +1 = +0.50 to +1.49, +2 = +1.50 to +2.49 and +3 = +2.50 to +3.49 [20].

### Measures of Physical Fitness

Three physical fitness components were evaluated using field tests from the Eurofit battery [21]. These tests are reliable and valid instruments for measuring fitness and are considered independent of one another [22].

*10 × 5 m Shuttle run test*: Two parallel lines (2 meters long) are drawn on the floor 5 m apart. On command, the subject ran as fast as possible from the starting line to the other line and returns to the starting line, crossing each line with both feet every time. The stopwatch was stopped when the participant crossed the end line with one foot. Results recorded to the nearest 0.1 s. The 10 × 5 m shuttle run test presents high reliability, and the ICC was calculated as 0.95 [23].

*Standing Broad Jump:* from a standing position immediately behind a line with feet approximately shoulder width apart, the adolescent jumped as far as possible with feet together. The rearmost foot was recorded as the final measure (cm.) of the jump. The swing of the upper limbs was permitted. The standing broad jump presents high reliability from test-retest analysis, and the ICCs reported a range from 0.94 to 0.95 [23]. and the coefficient of variation range was 7.3–11.9% [24].

*Sit-Ups*: the efficiency of abdominal musculature was measured by the maximum number of sit-ups (crunch) achieved within half a minute. The starting arrangement involved the participant lying on their back, fingers interlocked behind the nape, knees bent at a 90° angle and heels/feet flat on the floor. The participant was required to rise from the sitting position with the elbows straightened so that they touch the knees. The total number of sit-ups performed correctly within 30 s was counted and retained for analysis. The sit-ups test is a valid and reliable method to assess abdominal dynamic endurance and the ICC was 0.91 [25].

### Statistical analyses

Data analysis was performed using SPSS Statistics (IBM Corp. Released 2017. IBM SPSS Statistics for Windows, Version 25.0. Armonk, NY: IBM Corp). Preliminary ANOVA with 10 × 5 m as a dependent variable and sex and PHV as fixed factor it was performed, with Bonferroni post-hoc test. Stepwise linear regression was performed on the dataset to evaluate preliminary which factor influence change of direction (10 × 5 m test). 10 × 5 m as a dependent variable, and SBJ, SUP, Height, Body mass, and Leg length as independent variables. The reliability of the regression models was expressed with adjusted R Square and standard error of estimate. Data was linearized with a log transformation (Ln = loge). Analysis of covariance (ANCOVA) was used to evaluate the influence of sex (boys, girls); and PHV [19, 20]. as fixed factor on 10 × 5 m (as predictor of change of direction performance). Further, SBJ (as predictor of lower limb strength), SUP (as predictor of trunk strength), height, body mass, and leg length as covariates on 10 × 5 m. The significance level was set at *P* < 0.05.

## RESULTS

### Preliminary

**FIG. 1:**
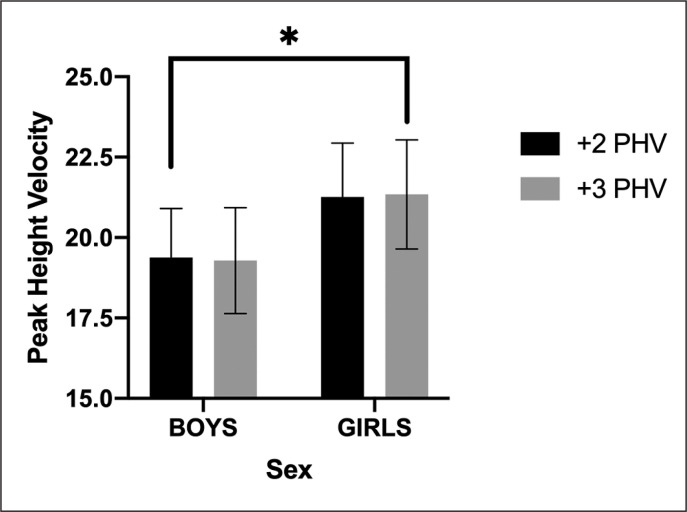
The trend for both sexes between the peak height velocity in 10x5m performance.

Preliminary analysis with 10 × 5 m as a dependent variable and sex and PHV as fixed factor, suggesting a significative difference between sex (p < 0.001) but not with PHV (p = 0.986) and interaction PHV × sex (p = 0.836) (Figure 1).

The Bonferroni post-hoc within PHV suggested a significant difference between +1 PHV and +2 PHV (p < 0.001), and between +1 PHV and +3 PHV (p < 0.001), but not between +2 PHV and +3 PHV (p = 0.962).

Preliminary stepwise linear regression results are presented in Table 1.

**TABLE 1:** Data from stepwise linear regression declared among both boys and girls, only boys, and only girls.

Model	R	R Square	Adj R Square	Std. Er of the Estimate	R Square Change	F Change	df1	df2	Sig. F Change
**Boys, Girls**									
SBJ	0.579	0.335	0.334	1.573	0.335	385.309	1.000	766	0.000
SBJ, Leg length	0.665	0.443	0.441	1.441	0.108	148.563	1.000	765	0.000
SBJ, Leg length, SUP	0.689	0.474	0.472	1.401	0.031	45.617	1.000	764	0.000

**Boys**									
SBJ	0.504	0.254	0.252	1.382	0.254	134.688	1.000	396	0.000
SBJ, SUP	0.520	0.271	0.267	1.368	0.017	9.139	1.000	395	0.003
SBJ, SUP, Leg Length	0.527	0.278	0.273	1.362	0.008	4.094	1.000	394	0.044

**Girls**									
Leg Length	0.518	0.268	0.266	1.468	0.268	134.77	1.000	368	0.000
Leg Length, SUP	0.572	0.327	0.324	1.409	0.059	32.437	1.000	367	0.000

Note. The models are ordered by adjusted R square value from smallest to largest.

Adj R Square = Adjusted R square; Std. Er of the Estimate = Standard Error of the Estimate; df = degree of freedom; SBJ = Standing Broad Jump; SUP = Sit-Up Test;

### Boys and Girls

**FIG. 2:**
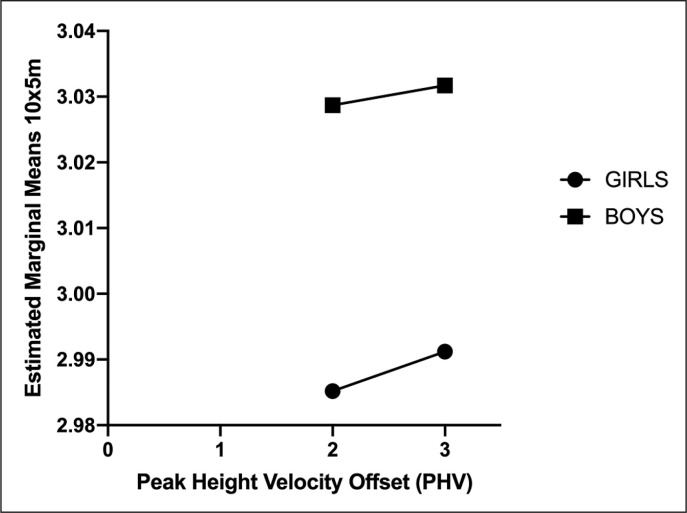
Estimated marginal means of 10x5m performance between sexes and the peak height velocity.

Trend of both sexes per PHV are visually represented in Figure 2. The model involved sex and PHV as fixed factors, and Height, Body mass, Leg length, SBJ, and SUP as covariates showed an adjusted R square of 0.473.

The output from ANCOVA (B parameter) of Body mass (-0.008), SBJ (-0.232), Leg length (-0.315), SUP (-0.081) and PHV2 (-0.005) are negative, suggesting that these parameters negatively influenced the performance. Only Height (0.579) was positive.

### Girls

**FIG. 3:**
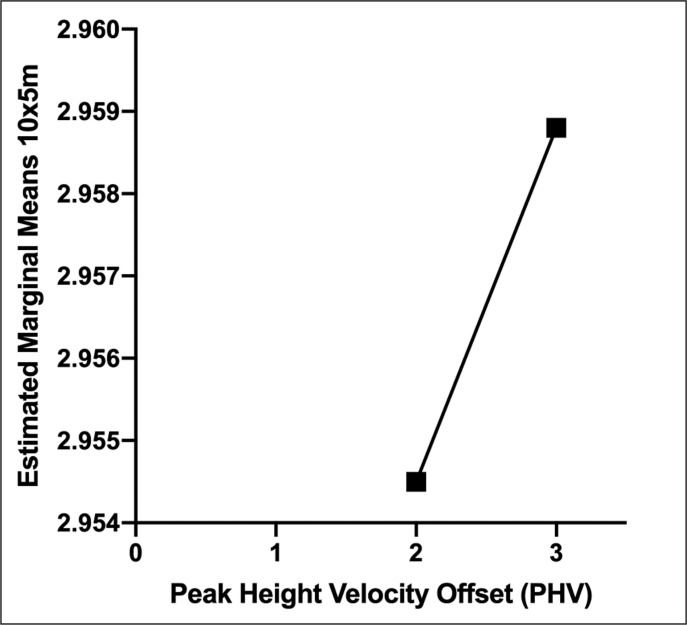
Estimated marginal means of 10x5m performance between the peak height velocity only for girls.

Trend per PHV is shown in Figure 3.

The model involved PHV as fixed factor, and Height, Body mass, Leg length, SBJ, and SUP as covariates showed an adjusted R square of 0.328

The output from ANCOVA (B parameter) of Body mass (-0.018), Height (-0.021), SBJ (-0.214), Leg length (-0.06), SUP (-0.113), and PHV2 (-0.008) was negative, suggesting that all the outcomes negatively influenced the performance in girls.

### Boys

**FIG. 4:**
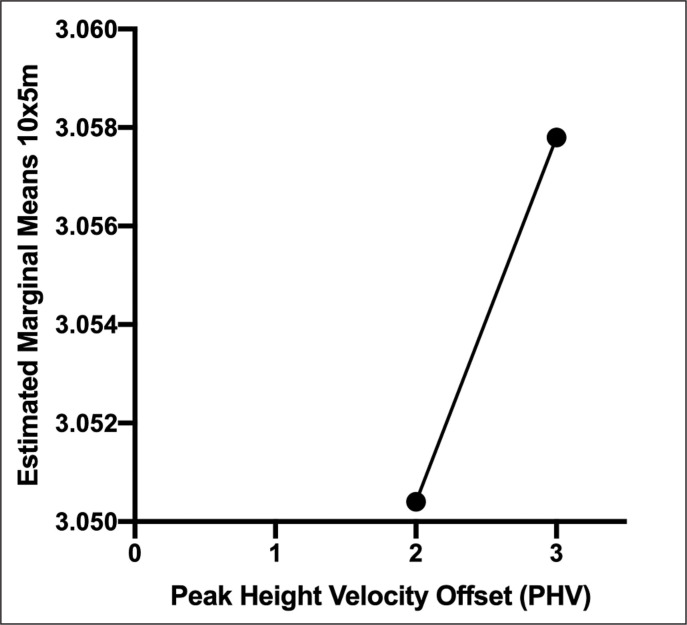
Estimated marginal means of 10x5m performance between the peak height velocity only for boys.

Trend per PHV is shown in Figure 4.

The model involved PHV as fixed factor, and Height, Body mass, Leg length, SBJ, and SUP as covariates showed an adjusted R square of 0.271

The output from ANCOVA (B parameter) of Height (-0.188), Leg length (-0.324), SUP (-0.04) and PHV2 (-0.004) was negative, suggesting that these parameters negatively influenced the performance. Body mass (0.016), SBJ (0.183), was positive.

## DISCUSSION

In sports, COD ability is a common outcome measure used to identify talented young athletes and monitor changes in performance [1]. Therefore, the purpose of this investigation was to assess the influence of anthropometric measures and maturation status on COD ability in adolescent males and females when these key variables can have a profound impact on this key fitness quality.

The results of this investigation suggest that repeated COD performance was influenced by anthropometrics, maturation and muscle qualities in adolescent boys and girls. In fact, the results of the current study demonstrated that in the female cohort all outcome variables negatively influenced the COD performance. However interestingly, lower limb strength as measured by the standing broad jump and body mass positively affected the COD performance in the male cohort. Considering these results, anthropometric characteristics and maturity offset affect COD performance and are different in girls and boys. The COD performance in girls appears to be negatively affected by maturation compared to boys. This result may be linked to the different hormonal responses during growth and maturation between adolescent males and females [26]. More specifically, the decreased performance in COD ability observed in females may be caused by an increase in body mass that is not adequately balanced by an increase in lower limb muscle strength and power [27].

The maturation status of the participant as measured by PHV was shown to influence repeated COD ability. This influence was demonstrated to be greater in the period immediately post PHV (PHV+1 to PHV+2) compared to later in maturation. This finding is consistent with current youth athlete development models [28] whereas a spurt in growth is not necessarily considered a positive influence on adolescent athlete’s performance. In fact, a faster maturation in youth when not adequately supported by correct training of physical qualities could become a negative influence on future performance. For these reasons, proper assessment of maturation provides valuable information that can be utilized by specialist sports coaches, strength and conditioning coaches and Physical Education teachers to inform the design of training programs to enhance COD ability.

Within the Youth Athlete Development Model [11] “agility” which is underpinned by COD ability is proposed to have an optimal window of opportunity immediately following PHV and can be trained at any point during the developmental process. In first instance, during this phase of development, there are rapid increases in size and weight with reported improvements in physical performance primarily as a result of changes in the hormones and maturation (24). Furthermore, Negra et al. [29], suggested that maturity status is one of the more significant predictors of COD ability when measured via the 505 test. The results from the current study confirm these findings, adolescents with a greater maturity status (PHV+3) performed better than their counterparts at a lower maturation level [30, 31]. Indicating that a maturation level corresponding with PHV+2 could be the optimal window of opportunity to train to change of direction (180°) performance.

Regarding anthropometric outcome measures from the current study, leg length negatively influenced COD performance. This negative relationship indicates a faster time to completion, most likely because longer legs foster shorter time to complete in the 10 × 5 m test. Similar results have recently been published by Negra and colleagues [29] who reported a significant association between leg length and COD performance in a large cohort of young soccer players. However, the COD test used in the study by Negra et al. [29] was a single 5-0-5 test. The influence of leg length on a test, such as the 10 × 5 shuttle run, is likely to be exponential because of the increased number of directional changes. In the light of this, Bourgeois et al. [32], suggested that during a change of direction task or test, the athlete is required to rapidly lower the mass center to obtain a proper force production. This is a benefit for shorter athletes (low leg length), as they generally have a lower mass center than athletes with long legs.

The results of the current investigation indicate that increased muscle power measured by the standing broad jump and muscle endurance measured by the sit-up test relate to shorter times in the 10 × 5 test. Demonstrating that these muscular qualities are important for performance in repeated COD tests. The nature of a 180° turns means the COD is strongly influenced by stretch shortening cycle performance and subsequent expression of power [33]. In fact, each 180° turn requires an acceleration phase from the beginning of a COD, a deceleration phase prior to the subsequent COD, the execution of a 180° turn and then a further acceleration. Previous research from de Hoyo et al. [34], has demonstrated that strength training targeting lower limb power is effective for increasing performance in COD tasks especially in adolescent with greater levels of maturations.

Of course, the results of this investigation must be considered alongside its limitation. The primary sports that the participants were competing in was not factored into the analysis. The sport a participant competes may influence their COD ability and future research should consider investigating this influence. Additionally, a standing broad jump can considered a superficial measure of an individual’s lower limb power. Whilst the results of this study demonstrated that lower limb power is important, a detailed force-velocity-power profile of the students was not possible due to the relatively large sample size and the applied nature of the study. More detailed kinetic and kinematic profiling of lower limb muscle function may provide diagnostic insight influence of strength and power on COD ability in adolescent males and females. The results of this study can inform the training prescription of programs targeting the improvement of COD ability in adolescent males and females. Practitioners designing these programs should target the development of lower limb strength and power and evaluate the maturation status of their athletes to ensure the timing is correct to maximize improvement in this important fitness quality.
